# *In Ovo* and Oral Administration of Probiotic Lactobacilli Modulate Cell- and Antibody-Mediated Immune Responses in Newly Hatched Chicks

**DOI:** 10.3389/fimmu.2021.664387

**Published:** 2021-04-12

**Authors:** Mohammadali Alizadeh, Jegarubee Bavananthasivam, Bahram Shojadoost, Jake Astill, Khaled Taha-Abdelaziz, Nadiyah Alqazlan, Nitish Boodhoo, Janan Shoja Doost, Shayan Sharif

**Affiliations:** ^1^ Department of Pathobiology, Ontario Veterinary College, University of Guelph, Guelph, ON, Canada; ^2^ Department of Pathology and Molecular Medicine & McMaster Immunology Research Centre, M. G. DeGroote School of Medicine, McMaster University, Hamilton, ON, Canada; ^3^ Quality Control Department, Artemis Technologies Inc., Guelph, ON, Canada; ^4^ Department of Pathology, Faculty of Veterinary Medicine, Beni-Suef University, Beni-Suef, Egypt

**Keywords:** lactobacilli, chicken, cytokine, lymphocyte, antibody, immune response

## Abstract

There is some evidence that lactobacilli can strengthen the immune system of chickens. This study evaluated the effects of *in ovo* and oral administration of a lactobacilli cocktail on cytokine gene expression, antibody-mediated immune responses, and spleen cellularity in chickens. Lactobacilli were administered either *in ovo* at embryonic day 18, orally at days 1, 7, 14, 21, and 28 post-hatches, or a combination of both *in ovo* and post-hatch inoculation. On day 5 and 10 post-hatch, spleen and bursa of Fabricius were collected for gene expression and cell composition analysis. On days 14 and 21 post-hatch, birds were immunized with sheep red blood cells (SRBC) and keyhole limpet hemocyanin (KLH), and sera were collected on days 7, 14, and 21 post-primary immunization. Birds that received lactobacilli (10^7^ CFU) *via* in *ovo* followed by weekly oral administration showed a greater immune response by enhancing antibody responses, increasing the percentage of CD4^+^ and CD4^+^CD25^+^ T cells in the spleen and upregulating the expression of interferon (IFN)-α, IFN-β, interleukin (IL)-8, IL-13, and IL-18 in the spleen and expression of IFN-γ, IL-2, IL-6, IL-8, IL-12, and IL-18 in the bursa. These findings suggest that pre-and post-hatch administration of lactobacilli can modulate the immune response in newly hatched chickens.

## Introduction

In commercial poultry production, newly hatched chicks may be held off feeding because of the variation in hatching time, and this might lead to feed deprivation for up to 72 hours in early hatched chicks (1). However, there is some evidence that delaying access to feed and water can adversely impact performance and immune responses in neonatal chickens (2). For example, delayed access to feed and water limits the development of lymphoid organs which subsequently affects the frequency of circulating lymphocytes and delays antibody mediated response following vaccination (3, 4). Furthermore, post-hatch environmental stressors such as hatchery processing, treatments and vaccine administration, beak trimming, transportation, heat, and high stocking density can negatively influence the immune system of newly hatched chicks, making them susceptible to pathogens (5, 6).

Antibiotic growth promoters (AGPs) have been used widely in the poultry industry to control infectious disease, improve performance, and minimize the negative effects associated with post-hatch environmental stressors (7). However, the use of AGPs is gradually being phased out because of concerns associated with emergence of antimicrobial resistance microorganisms as well as antibiotic residues in animal products (8, 9). Several feed additives have been introduced to the poultry industry as a potential alternative for AGP; among them, probiotics have gain significant attention because of their ability to confer health benefits to the birds (10, 11). Lactobacilli are considered as the most common probiotics and natural inhabitants of the chicken intestinal tract (12). These bacteria exert their intestinal health benefits through different mechanisms such as competitive exclusion, antimicrobial activities, maintenance of intestinal barrier functions, and modulation of the immune system (13, 14). In this context, we have previously demonstrated that probiotics are able to modify the expression of a wide array of genes involved in immune induction of different subsets of the immune system such as cytokines (interferon (IFN)-γ, interleukin (IL)-12, IL-13 and IL-6) that can directly or indirectly enhance serum IgM and IgY antibody production in response to antigens (15, 16).

Probiotics can be administered through different methods such as feed, water, and spray (17). *In ovo* delivery of selected probiotics into the amniotic sac of embryonated eggs, is a candidate route that may facilitate early colonization of beneficial bacteria in the intestine and alleviate the adverse effects associated with environmental stressors and delayed access to feed and water (18). Interaction between the gut microbiome and the host immune system plays a critical role in the development of immunity to invading microbes (19). Some studies have shown that early colonization of the intestine with beneficial bacteria can modulate the immune system of chickens and modify the gut microbiota composition by reducing the colonization of pathogens in the intestine (20–23). It has also been demonstrated that continuous inoculation of probiotics is required to sustain intestinal colonization and extend their health benefits (24). Therefore, the present study was undertaken to evaluate and compare one time *in ovo* inoculation of a selected lactobacilli mixture versus weekly oral administration, and the combination of both delivery methods on innate and adaptive immune responses in chickens.

## Materials and Methods

### Experimental Animals and Housing

Two hundred and forty embryonated commercial broiler chicken eggs were obtained from the Arkell Poultry Research Station, University of Guelph. Embryonated eggs were incubated at recommended temperature and relative humidity. Post-hatch, day old chicks were group housed according to treatment in separate floor pens (30 birds per pen) at Arkell Poultry Research. The research was approved by the University of Guelph Animal Care Committee according to the guidelines of the Canadian Council on Animal Care.

### Bacterial Strains and Culture Conditions

Wildtype *Lactobacillus* spp (*L. salivarius-JB/SL-26*, *L. johnsonii-JB/SL-39, L. reuteri-JB/SL-42*, and *L. crispatus-JB/SL-44*), were previously isolated in our lab. *Lactobacillus* strains were genotyped and characterized accordingly (25, 26). Throughout this study, all *Lactobacillus* isolates were cultured in MRS broth (Gibco, Ca) and maintained under anaerobic conditions (37 °C and no shaking) until required. Bacteria quantification (colony forming unit; CFU/ml) was performed by 10-fold serial dilution on MRS agar under anaerobic conditions (37 °C and no shaking) for purposes of preparing and confirming inoculums. Overnight *Lactobacillus* spp cultures were washed (4000 rpm for 10 min) and resuspended in phosphate-buffered saline (PBS). Bacterial inoculums, equal parts mixture of individual strains (10^7^ CFU/ml) were prepared in PBS and kept on ice until required for inoculations. Lactobacilli used in the present study have been isolated from the intestines of healthy broiler chickens (unpublished data). Therefore, these bacteria are considered as members of the chicken intestinal microbiome.

### Experimental Design

*In ovo inoculations*: On embryonic day 18 (ED18), eggs were disinfected with 70% ethanol and a hole was punched into the shell with a 23-gauge needle. Eggs were randomly assigned to each experimental group. Eggs were injected with 100 μL of a cocktail of *Lactobacillus* spp (10^6^ or 10^7^ CFU/100 μl/egg; (*L. salivarius-JB/SL-26*, *L. johnsonii-JB/SL-39, L. reuteri-JB/SL-42*, and *L. crispatus-JB/SL-44*), or PBS (lactobacilli diluent) into the amniotic sac using a 23-gauge 2.5 cm needle (21). Each *Lactobacillus* strain was grown separately and prepared at the certain dose in PBS. The multi-strain cocktail was prepared by mixing equal amounts of each strain. Untreated group (no injections) was used as negative control. Following *in ovo* injections, eggs were allocated into 8 experimental groups summarized in **Table 1**. All eggs were incubated in the same incubator and the same hatchery in Arkell Research Station, University of Guelph.

**Table 1:** Experimental groups.

Group	Abbreviated Names	*In ovo* Injection (ED18)^1^	Oral Lactobacilli Administration (days 1, 7, 14, 21, 28 post-hatch)
1	*In ovo* 10^6^	10^6^ CFU Lactobacilli/100 µl/egg	None
2	*In ovo* 10^7^	10^7^ CFU Lactobacilli/100 µl/egg	None
3	*In ovo* 10^6^ + Gav 10^6^	10^6^ CFU Lactobacilli/100 µl/egg	10^6^ CFU Lactobacilli/ml
4	*In ovo* 10^7^ + Gav 10^7^	10^7^ CFU Lactobacilli/100 µl/egg	10^7^ CFU Lactobacilli/ml
5	Gav^2^ 10^6^	None	10^6^ CFU Lactobacilli/ml
6	Gav 10^7^	None	10^7^ CFU Lactobacilli/ml
7	PBS^3^	PBS/100 µl/egg	None
8	UN^4^	None	None

^1^Embryonic day 18.

^2^Gavage.

^3^Phosphate buffered saline.

^4^Untreated.

### Immunization and Sample Collection

Immunization and sample collection was performed as described previously (21). On days 14 and 21 post-hatch, birds were immunized intramuscularly with 0.25 ml of 2% sheep red blood cells (SRBC) (PML Microbiologicals, Mississauga, ON, CAN) and 100 μg of keyhole limpet hemocyanin (KLH) (Sigma-Aldrich, Oakville, ON, CAN) in 0.25 ml PBS. The untreated group were inoculated intramuscularly with 0.5 ml of PBS. Blood samples were collected from the wing vein of 12 birds per treatment group on days 0, 7, 14, and 21 post primary immunization. Blood samples were kept at room temperature (RT) for two hours (hrs) and centrifuged at 580 × g for 10 mins for serum separation. Serum samples were collected and stored at −20°C for antibody analysis. On days 5 and 10 post-hatch, 6 birds per treatment were euthanized and the bursa of Fabricius and spleen were collected, kept in RNAlater (Invitrogen, Burlington, ON, CAN) and stored at −80°C for gene expression analysis. Splenic tissues were also collected in 1*X* Hanks’ balanced salt solution (HBSS) (*Gibco, Grand Island, NY*) and stored on ice.

### Spleen Mononuclear Cells Preparation and Flow Cytometry Analysis

Mononuclear cells were prepared from the spleens of 6 birds per treatment group as previously described (21). Briefly, tissue samples were rinsed with HBSS and passed through a 40-μm nylon cell strainer using the flat end plunger of a 1-ml syringe in 5 ml complete RPMI (Invitrogen, Burlington, ON, CAN) medium (10% fetal bovine serum and 1% Penicillin-Streptomycin: Gibco, Grand Island, NY). Cell suspension were subsequently overlaid on 4 ml Histopaque-1077 (Sigma, Oakville, ON) for density gradient separation (400 g for 20 min). The white buffy coat at the interface were harvested and washed twice with RPMI medium. Mononuclear cells were counted using an automated cell counter MOXI Z (Orflo, Ketchum, ID, USA) after which 100 µl of mononuclear cell suspension, from each group was seeded in round bottom 96-well plates at a density of 5 x 10^5^ cells/well in RPMI medium.

Mononuclear cells were washed twice with flow cytometry staining buffer (FACS buffer; PBS containing 1% bovine serum albumin) and stained for 30 min at 4^°^C in the dark with fluorescent monoclonal antibodies consisting of two different surface staining panels. Panel 1: mouse anti-chicken CD3-PB, mouse anti-chicken CD4-PE-Cy7, mouse anti-chicken CD8-APC, and human anti-chicken CD25-FITC. Panel 2: mouse anti-chicken Bu-1-PB, mouse anti-chicken IgM-APC-Cy7, and mouse anti-chicken monocyte/macrophage-FITC (KUL01). All monoclonal antibodies were purchased from SouthernBiotech (SouthernBiotech, Birmingham, AL, USA) except CD25-FITC purchased from Bio-Rad (Mississauga, ON, CAN). For both staining panels, the fixable Live/Dead near- Infrared fluorescent reactive dye (Thermo Fisher Scientific, CAN) was used for dead cell exclusion. Following staining, cells were washed twice in FACS buffer, fixed in 2% paraformaldehyde. Fixed mononuclear cells were acquired on a FACS Canto II flow cytometer (BD Bioscience, San Jose, CA, USA), and data were analyzed using FlowJo Software (v.10).

### Serological Analysis

**(i) SRBC-Specific Antibodies:** A direct hemagglutination assay was performed for detection of antibody responses against SRBC in sera, as previously described (15). Serum samples were heat-treated at 56^°^C for 30 min, then 50 μl of PBS containing 0.05% bovine serum albumin was added into each well of a 96-well round-bottom microplate, and two-fold serial dilutions of serum samples were generated in duplicates. Subsequently, 50 μl of 1% SRBC in PBS was added to each well, and plates were shaken for 1 min, followed by incubation (24 hrs at 37^°^C). The results were considered positive when at least 50% of SRBC agglutination was observed.

**(ii) KLH-Specific Antibodies**: Detection of KLH-specific IgG (IgY) and IgM titers in serum samples was performed by indirect enzyme-linked immunosorbent assay (ELISA) as previously described (16, 21). Briefly, each well of a 96-well flat-bottomed Maxisorp high binding microplate was coated with 1 μg/ml KLH in 100 μl of coating buffer (0.1 M NaHCO_3_, pH 9.6 with 30 μg/ml BSA) and incubated overnight at 4^°^C. Plates were washed 4 times with PBST (0.05% Tween 20; P137 Sigma-Aldrich Inc., St. Louis, MO) and subsequently, incubated (2 hrs at RT in the dark) in 200 μl/well blocking buffer (PBST containing 0.25% of fish skin; Sigma-Aldrich, Oakville, ON). Plates were again washed 4 times with PBST and incubated (2 hrs at RT in the dark) with 100 μl/well of chicken sera (diluted 1:200 v/v in blocking buffer). Plates were washed again (4 times in PBST), prior to incubation (1 hr at RT in the dark) in 100 μl/well of detection antibody (goat anti-chicken IgY-Fc and IgM-Fc, Bethyl laboratories) conjugated with horseradish peroxidase (diluted at 1/5000 in blocking buffer). Following a final wash (4 times in PBST), plates were developed with 100 μl/well horseradish peroxidase substrate ABTS (2,2’-Azino-bis (3-ethylbenzothiazoline-6-sulfonic acid; Mandel Scientific, Guelph, ON, CAN). Absorbance was measured at 405 nm using the microplate reader (Epoch, BioTek Instruments Inc., Winooski, VT) within 30 min of ABTS addition. Positive and negative control sera (fetal bovine serum) were included in each plate to normalize plate-to-plate variation. Sample/positive (Sp) ratios were calculated according to the following formula: (mean of test sample - mean of negative control)/(mean of positive control - mean of negative control).

### RNA Extraction and cDNA Synthesis

RNA extraction and reverse transcription were performed as previously described (21, 27). Total RNA was extracted from spleen and bursa of Fabricius mononuclear cells using TRIzol^®^ (Invitrogen, Carlsbad, CA, USA) according to the manufacturer’s recommendations. Total RNA was treated with DNase (DNA-free kit, Ambion, Austin, TX) and quantity and purity of RNA samples were measured by a Nanodrop spectrophotmeter (Thermo Scientific, Wilmington, DE). Reverse-transcription to complementary DNA (cDNA) was performed using Superscript^®^ II First-Strand Synthesis kit (Invitrogen) according to the manufacturer’s protocol.

### Quantitative Real-Time PCR (qRT-PCR)

qRT-PCR was performed using LightCycler^®^ 480 II system (Roche Diagnostics GmbH, Mannheim, DE) as described previously (21, 27). Each reaction consisted of 10 μl of 2X SYBR Green Master Mix (Roche Diagnostics), 1 μl of forward and 1 μl of reverse primers (5 μM), 3 μl PCR-grade water and 5 μl of cDNA (1:10, diluted in nuclease free-water). The qRT-PCR cycling protocol included an initial denaturation step at 95°CC, followed by amplification for 40-50 cycles consisting of 95°CC for 10 sec, annealing temperature (**Table 2**), and extension at 72°CC for 10 sec. All primers used in this study (**Table 2**) were synthesized by Sigma-Aldrich (Oakville, ON).

**Table 2:** Primer sequences used for real-time quantitative PCR^1^.

Gene^2^	Primer sequence^3^ (5’-3’)	Annealing temperature	GeneBank accession number
*IFN-α*	F: ATCCTGCTGCTCACGCTCCTTCT R: GGTGTTGCTGGTGTCCAGGATG	64	AB021154
*IFN*-*β*	F: GCCTCCAGCTCCTTCAGAATACG R: CTGGATCTGGTTGAGGAGGCTGT	64	AY974089
*IFN-γ*	F: ACACTGACAAGTCAAAGCCGCACA R: AGTCGTTCATCGGGAGCTTGGC	60	X99774
*IL-1β*	F: GTGAGGCTCAACATTGCGCTGTA R: TGTCCAGGCGGTAGAAGATGAAG	64	Y15006
*IL-2*	F: TGCAGTGTTACCTGGGAGAAGTGGT R: ACTTCCGGTGTGATTTAGACCCGT	60	NM_204153.2
*IL-6*	F: CGTGTGCGAGAACAGCATGGAGA R: TCAGGCATTTCTCCTCGTCGAAGC	60	NM_204628.1
*IL-8*	F: CCAAGCACACCTCTCTTCCA R: GCAAGGTAGGACGCTGGTAA	64	AJ009800
*IL-12p40*	F: CCAAGACCTGGAGCACACCGAAG R: CGATCCCTGGCCTGCACAGAGA	60	AY262752.1
*IL-13*	F: ACTTGTCCAAGCTGAAGCTGTC R: TCTTGCAGTCGGTCATGTTGTC	60	AJ621250.1
*IL-18*	F: GAAACGTCAATAGCCAGTTGC R: TCCCATGCTCTTTCTCACAACA	64	AY628648.2
*TGF-β^4^*	F: CGGCCGACGATGAGTGGCTC R: CGGGGCCCATCTCACAGGGA	60	M31160.1
*β-Actin*	F: CAACACAGTGCTGTCTGGTGGTA R: ATCGTACTCCTGCTTGCTGATCC	58	X00182

^1^The listed oligonucleotides were used to analyze gene expression via real-time quantitative PCR.

^2^IFN, Interferon; IL, Interleukin.

^3^F, forward; R, reverse.

^4^TGF-β, transforming growth factor beta.

### Statistical Analysis

The expression levels of all genes were calculated relative to the housekeeping gene (β-actin) using the LightCycler^®^ 480 software (Roche Diagnostics), and data were analyzed using the generalized linear model (GLM) procedure of SAS (SAS Institute Inc., Cary, NC). Differences among treatments means were determined using Tukey’s multiple comparison test after log transformation when error deviations did not have homogenous variance across the treatments. A *P*-value of <0.05 was considered statistically significant.

## Results

### Hatchabilit*y*

Embryonated eggs were inoculated *via* the amniotic sac at ED18 with either a lactobacilli cocktail or PBS. Here we report that *in ovo* inoculation with a live lactobacilli cocktail did not affect embryo development. Moreover, eggs inoculated with the highest dose at 10^7^ CFU of lactobacilli continued to develop normally as those inoculated with 10^6^ CFU of lactobacilli. All embryonated eggs were hatched confirming that neither lactobacilli inoculation nor PBS injection affected hatchability. The overall hatchability was estimated at 99.91%. Chicks from each group showed no adverse effects in their continued growth post hatch.

### Cytokine Gene Expression in Spleen

**Figure 1:**
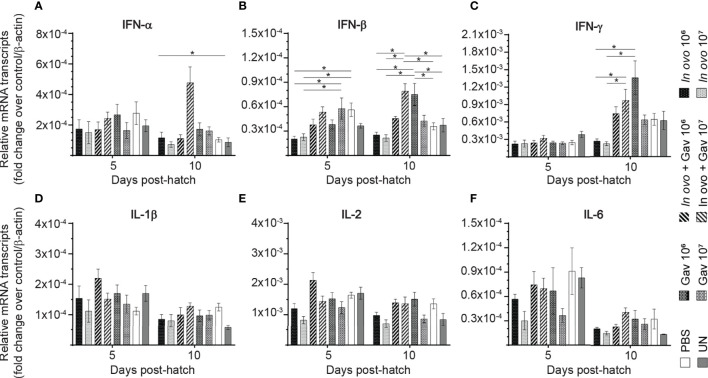
Relative gene expression in the spleen of chickens inoculated with lactobacilli. Spleen samples were collected at days 5 and 10 post-hatch from all treatment groups: groups 1 and 2 received 10^6^ and 10^7^ CFUs of lactobacilli cocktail *in ovo*, respectively, at embryonic day eighteen; groups 3 and 4 received 10^6^ and 10^7^ CFUs of lactobacilli, respectively, *via* both *in ovo* and oral gavage (on days 1, 7, 14, 21, 28 post-hatch); groups 5 and 6 received 10^6^ and 10^7^ CFUs of lactobacilli, respectively, *via* oral gavage; group 7 was served as a negative control group and was injected with phosphate-buffered saline (PBS); group 8 remained untreated (UN). Fold change was used to characterize differences in gene expression of IFN-α, IFN-β, IFN-γ, IL-1β, IL-2 and IL-6 **(A–F)**, in comparison to negative control group. Statistical significance among treatment groups was calculated using SAS Proc GLM (General Linear Model) followed by Tukey’s comparison test. Error bars represent standard errors of the mean. Results were considered statistically significant if *P* < 0.05. Bars with asterisks represent a significant difference among treatments. The data are representative samples from 6 individual birds.

**Figure 2:**
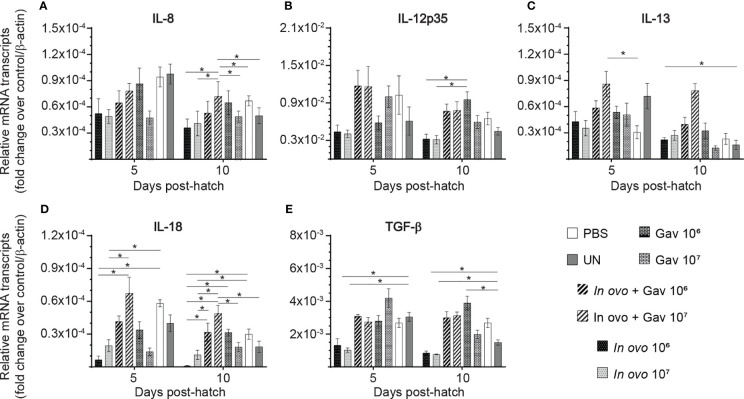
Relative gene expression in the spleen of chickens inoculated with lactobacilli. Spleen samples were collected at days 5 and 10 post-hatch from all treatment groups: groups 1 and 2 received 10^6^ and 10^7^ CFUs of lactobacilli cocktail *in ovo*, respectively, at embryonic day eighteen; groups 3 and 4 received 10^6^ and 10^7^ CFUs of lactobacilli, respectively, *via* both *in ovo* and oral gavage (on days 1, 7, 14, 21, 28 post-hatch); groups 5 and 6 received 10^6^ and 10^7^ CFUs of lactobacilli, respectively, *via* oral gavage; group 7 was served as a negative control group and was injected with phosphate-buffered saline (PBS); group 8 remained untreated (UN). Fold change was used to characterize differences in gene expression of IL-8, IL-12, IL-13, IL-18 and TGF-β **(A–E)**, in comparison to negative control group. Statistical significance among treatment groups was calculated using SAS Proc GLM (General Linear Model) followed by Tukey’s comparison test. Error bars represent standard errors of the mean. Results were considered statistically significant if *P* < 0.05. Bars with asterisks represent a significant difference among treatments. The data are representative samples from 6 individual birds.

The results for gene expression in spleen are presented in **Figures 1** and **2A, B**. Gene expression analysis revealed differential trends in most cytokines tested. *In ovo* and/or post-hatch oral inoculation with lactobacilli cocktail did not alter expression (P > 0.05) of interleukin (IL)-1β, IL-2, and IL-6 (**Figures 1D–F**) on days 5 and 10 post-hatch. Expression of interferon (IFN)-β (**Figure 1B**) on day 5, IFN-γ (**Figure 1C**) on day 10, IL-18 (**Figure 2D**), and transforming growth factor beta (TGF)-β (**Figure 2E**) on days 5 and 10 post-hatch were downregulated in birds that were inoculated *in ovo* with 10^6^ or 10^7^ CFU of lactobacilli compared to the PBS control group (P < 0.05). In contrast, bird inoculated with 10^7^ CFU of lactobacilli both *in ovo* and orally post-hatch had a significant up-regulation of IFN-α (**Figure 1A**), IFN-β (**Figure 1B**), and IL-13 (**Figure 2C**) on day 10 post-hatch compared to the PBS control group (P < 0.05). These results further demonstrate that expression of IFN-β (**Figure 1B**) was upregulated in birds that were only orally inoculated with 10^6^ CFU of lactobacilli cocktail on day 10- post-hatch (P < 0.05).

### Cytokine Gene Expression in the Bursa of Fabricius

**Figure 3:**
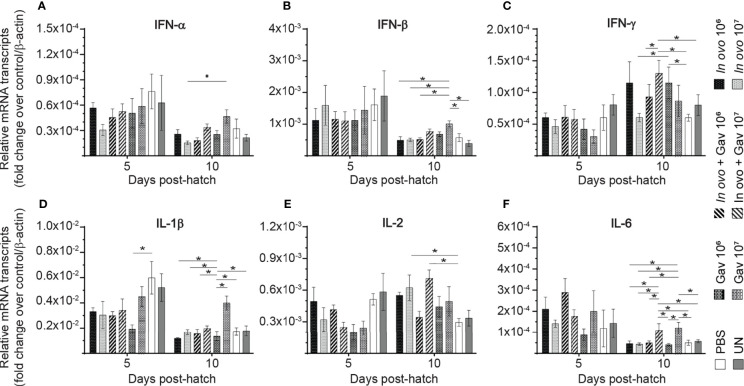
Relative gene expression in the bursa of Fabricius of chickens inoculated with lactobacilli. The bursa of Fabricius samples were collected at days 5 and 10 post-hatch from all treatment groups: groups 1 and 2 received 10^6^ and 10^7^ CFUs of lactobacilli cocktail *in ovo*, respectively, at embryonic day eighteen; groups 3 and 4 received 10^6^ and 10^7^ CFUs of lactobacilli, respectively, *via* both *in ovo* and oral gavage (on days 1, 7, 14, 21, 28 post-hatch); groups 5 and 6 received 10^6^ and 10^7^ CFUs of lactobacilli, respectively, *via* oral gavage; group 7 was served as a negative control group and was injected with phosphate-buffered saline (PBS); group 8 remained untreated (UN). Fold change was used to characterize differences in gene expression of IFN-α, IFN-β, IFN-γ, IL-1β, IL-2, and IL-6 **(A–F)**, in comparison to negative control group. Statistical significance among treatment groups was calculated using SAS Proc GLM (General Linear Model) followed by Tukey’s comparison test. Error bars represent standard errors of the mean. Results were considered statistically significant if *P* < 0.05. Bars with asterisks represent a significant difference among treatments. The data are representative samples from 6 individual birds.

**Figure 4:**
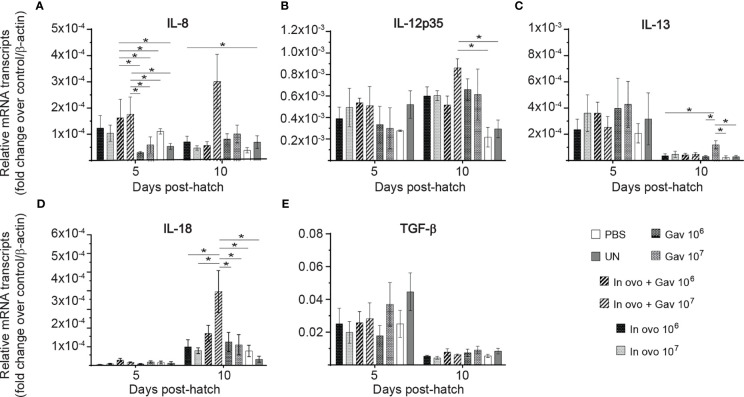
Relative gene expression in the bursa of Fabricius of chickens inoculated with lactobacilli. The bursa of Fabricius samples were collected at days 5 and 10 post-hatch from all treatment groups: groups 1 and 2 received 10^6^ and 10^7^ CFUs of lactobacilli cocktail *in ovo*, respectively, at embryonic day eighteen; groups 3 and 4 received 10^6^ and 10^7^ CFUs of lactobacilli, respectively, *via* both *in ovo* and oral gavage (on days 1, 7, 14, 21, 28 post-hatch); groups 5 and 6 received 10^6^ and 10^7^ CFUs of lactobacilli, respectively, *via* oral gavage; group 7 was served as a negative control group and was injected with phosphate-buffered saline (PBS); group 8 remained untreated (UN). Fold change was used to characterize differences in gene expression of IL-8, IL-12, IL-13, IL-18 and TGF-β **(A–E)**, in comparison to negative control group. Statistical significance among treatment groups was calculated using SAS Proc GLM (General Linear Model) followed by Tukey’s comparison test. Error bars represent standard errors of the mean. Results were considered statistically significant if *P* < 0.05. Bars with asterisks represent a significant difference among treatments. The data are representative samples from 6 individual birds.

Gene expression analysis in in the bursa of Fabircius (**Figures 3** and **4**) was performed to gain further insight into the possible mechanism by which lactobacilli may modulate the early development of B cells in the chicken. The results demonstrate that expression of TGF-β (**Figure 4E**) was not affected by lactobacilli treatment (P > 0.05). Post-hatch oral inoculation only with 10^6^ CFU of lactobacilli significantly increased expression of IFN-γ (**Figure 3C**) on day 10 post-hatch, when compared to the PBS control group (P < 0.05). Expression of IFN-β (**Figure 3B**), IL-1β (**Figure 3D**), IL-6 (**Figure 3F**) and IL-13 (**Figure 4C**) on day 10 post-hatch were significantly up-regulated in birds that received 10^7^ CFUs of lactobacilli orally (P < 0.05). *In ovo* inoculation with 10^7^ CFUs of lactobacilli upregulated the expression of IL-2 (**Figure 3E**) on day 10 post-hatch compared to the PBS control group (P < 0.05). Combined administration of 10^6^ CFU of lactobacilli induced significantly higher expression of IL-8 (**Figure 4A**) on day 5 post-hatch (P < 0.05). Chicks that received 10^7^ CFU of lactobacilli both *in ovo* and orally post-hatch demonstrated significantly higher expression of IFN-γ (**Figure 3C**), IL-2 (**Figure 3E**), IL-6 (**Figure 3F**), IL-8 (**Figure 3G**), IL-12 (**Figures 4BH**), and IL-18 (**Figure 4D**) on day 10 post-hatch when compared to the PBS control group (P < 0.05).

### Macrophage and Lymphocyte Populations

**Figure 5:**
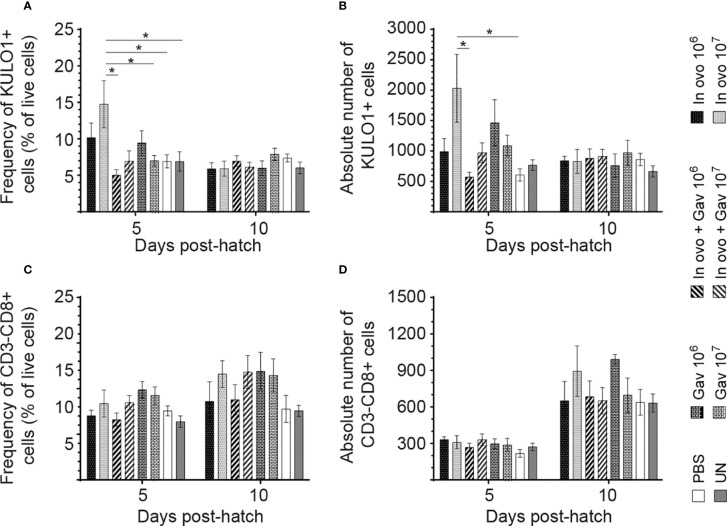
Changes in the frequency of spleen innate immune cells mediated by lactobacilli inoculation. Spleen samples were collected at days 5 and 10 post-hatch from all treatment groups: groups 1 and 2 received 10^6^ and 10^7^ CFUs of lactobacilli cocktail *in ovo*, respectively, at embryonic day eighteen; groups 3 and 4 received 10^6^ and 10^7^ CFUs of lactobacilli, respectively, *via* both *in ovo* and oral gavage (on days 1, 7, 14, 21, 28 post-hatch); groups 5 and 6 received 10^6^ and 10^7^ CFUs of lactobacilli, respectively, *via* oral gavage; group 7 was served as a negative control group and was injected with phosphate-buffered saline (PBS); group 8 remained untreated (UN). Spleen mononuclear cells were stained and flow cytometric analysis was performed to determine the respective frequency and absolute numbers of monocyte/macrophages (KUL01^+^; **A, B**) and CD3^-^CD8^+^ T cells **(C, D)**. Statistical significance among treatment groups was calculated using SAS Proc GLM (General Linear Model) followed by Tukey’s comparison test. Error bars represent standard errors of the mean. Results were considered statistically significant if *P* < 0.05. Bars with asterisks represent a significant difference among treatments. The data are representative samples from 6 individual birds.

**Figure 6:**
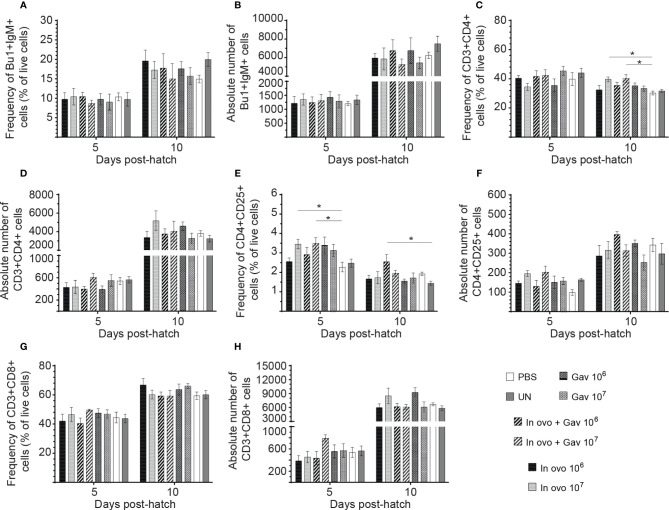
Changes in the frequency of spleen lymphocytes cells mediated by lactobacilli inoculation. Spleen samples were collected at days 5 and 10 post-hatch from all treatment groups: groups 1 and 2 received 10^6^ and 10^7^ CFUs of lactobacilli cocktail *in ovo*, respectively, at embryonic day eighteen; groups 3 and 4 received 10^6^ and 10^7^ CFUs of lactobacilli, respectively, *via* both *in ovo* and oral gavage (on days 1, 7, 14, 21, 28 post-hatch); groups 5 and 6 received 10^6^ and 10^7^ CFUs of lactobacilli, respectively, *via* oral gavage; group 7 was served as a negative control group and was injected with phosphate-buffered saline (PBS); group 8 remained untreated (UN). Spleen mononuclear cells were stained and flow cytometric analysis was performed to determine the respective frequency and absolute numbers of B cells (Bu-1^+^ IgM^+^;**A, B**) and **(C)** T cell subsets (CD3^+^CD4^+^, CD3^+^CD4^+^CD25^+^, CD3^+^CD8^+^; **C–H**). Statistical significance among treatment groups was calculated using SAS Proc GLM (General Linear Model) followed by Tukey’s comparison test. Error bars represent standard errors of the mean. Results were considered statistically significant if *P* < 0.05. Bars with asterisks represent a significant difference among treatments. The data are representative samples from 6 individual birds.

Results for the flow cytometric analysis of macrophages and CD3^-^CD8^+^ T cells are presented in **Figure 5**. Birds that received 10^7^ CFU of lactobacilli *in ovo* had higher frequency and absolute numbers of macrophages compared to the PBS control group (**Figures 5A, B**) at day 5 post-hatch (P < 0.05). However, frequency and absolute numbers of CD3^-^CD8^+^ T cell subsets were not affected by treatment groups. Results for the flow cytometric analysis of B cell (IgM^+^) and T cell subsets (CD4^+^, CD4^+^CD25^+^ and CD8^+^) data are presented in **Figure 6**. The Bu-1^+^IgM^+^ B cell subsets (**Figures 6A, B**) and CD3^+^CD8^+^ T cell (**Figures 6G, H**) and were not affected by any of the treatments at both time points (P > 0.05). However, birds that received 10^7^ CFU of lactobacilli *in ovo* only or both *in ovo* and orally had higher frequency of CD3^+^CD4^+^ T cells (**Figure 6C**) on day 10 post-hatch; and increased the frequency of CD4^+^CD25^+^ T regulatory (Treg) cells (**Figure 6E**) on day 5 post-hatch, when compared to the PBS control group (P < 0.05). In addition, higher frequency of CD4^+^CD25^+^ Treg cells (**Figure 6E**) was observed in birds that received 10^6^ CFU of lactobacilli both *in ovo* and orally post-hatch compared to the untreated birds on day 10 post-hatch (P < 0.05).

### Antibody-Mediated Immune Responses

#### Anti-SRBC Response

**Figure 7:**
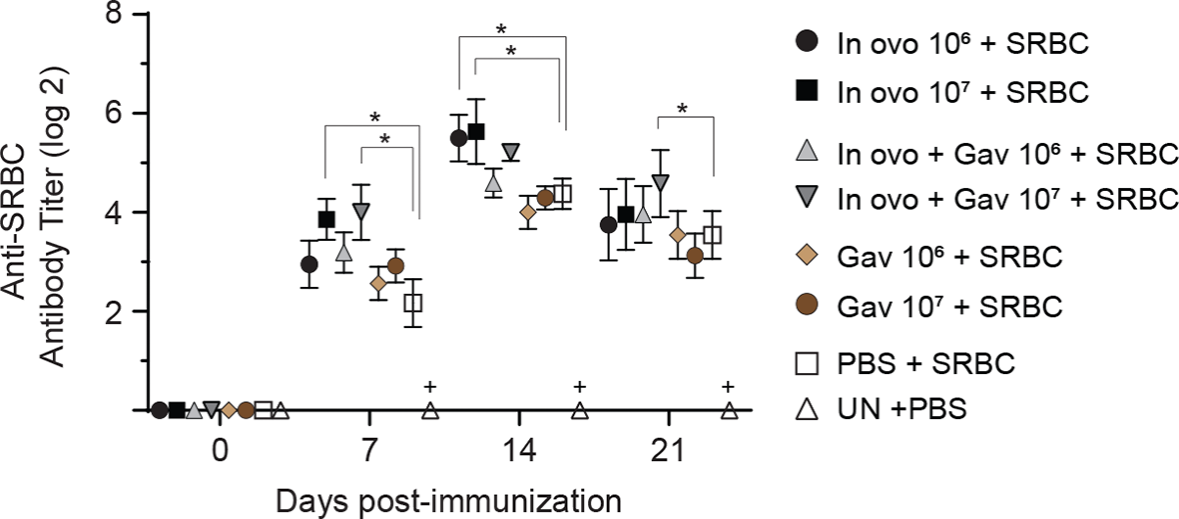
Serum anti-SRBC antibody titers as determined by direct hemagglutination assay. Serum samples were collected post-hatch from chickens immunized with SRBC and pre-treated with a lactobacilli cocktail as follow: groups 1 and 2 received 10^6^ and 10^7^ CFUs of lactobacilli cocktail *in ovo*, respectively, at embryonic day eighteen; groups 3 and 4 received 10^6^ and 10^7^ CFUs of lactobacilli, respectively, *via* both *in ovo* and oral gavage (on days 1, 7, 14, 21, 28 post-hatch); groups 5 and 6 received 10^6^ and 10^7^ CFUs of lactobacilli, respectively, *via* oral gavage; group 7 was injected with phosphate-buffered saline (PBS); group 8 remained untreated (UN). On days 14 and 21 post-hatch, birds were immunized intramuscularly with 2% SRBC in 0.25 ml of ml PBS. The untreated group (UN) was inoculated intramuscularly with 0.5 ml of PBS and served as the negative control. Statistical significance among treatment groups was calculated using SAS Proc GLM (General Linear Model) followed by Tukey’s comparison test. Error bars represent standard errors of the mean. Results were considered statistically significant if *P* < 0.05. Bars with asterisks represent a significant difference among treatments. The data are representative samples from 12 individual birds.

The production of antigen-specific antibodies represents a major defense mechanism of humoral immune response. The results for antibody-mediated immune responses against SRBC are presented in **Figure 7**. Higher antibody titers against SRBC were observed in all treatment groups when compared to the negative control group (non-treated and non-immunized birds, P < 0.05). *In ovo* inoculation of eggs with 10^7^ CFUs of lactobacilli significantly increased serum anti-SRBC antibody titers on days 7 and 14 post-primary immunization, while birds that received 10^7^ CFUs of lactobacilli *in ovo* and orally post-hatch showed higher titers of serum anti-SRBC antibodies on days 7 and 21 post-primary immunization when compared to the positive control group *(in ovo* injected with PBS and immunized with SRBC, P < 0.05).

#### Anti-KLH Response

**Figure 8:**
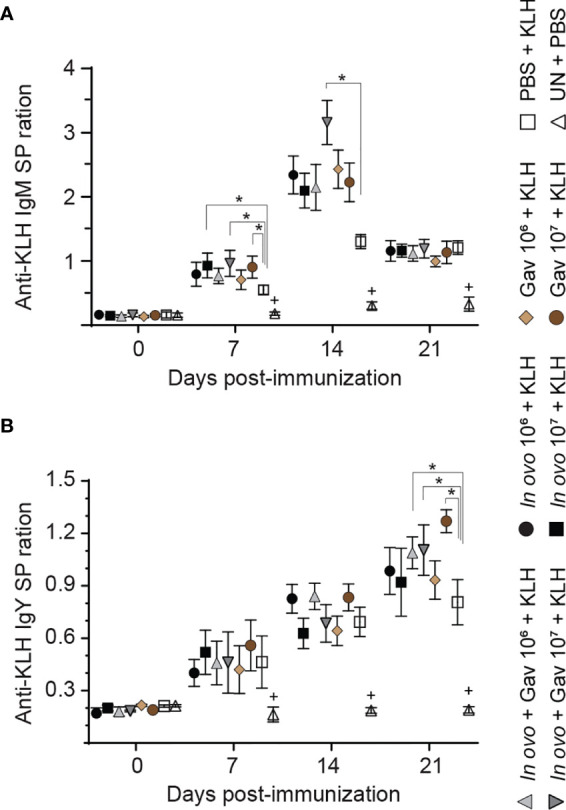
Serum anti-KLH antibodies titers as a measure of immune competence. Serum anti-KLH IgM **(A)** and IgY **(B)** titers were determined by indirect ELISA. Serum samples were collected post-hatch from chickens immunized with KLH and pre-treated with a lactobacilli cocktail as follow: groups 1 and 2 received 10^6^ and 10^7^ CFUs of lactobacilli cocktail *in ovo*, respectively, at embryonic day eighteen; groups 3 and 4 received 10^6^ and 10^7^ CFUs of lactobacilli, respectively, *via* both *in ovo* and oral gavage (on days 1, 7, 14, 21, 28 post-hatch); groups 5 and 6 received 10^6^ and 10^7^ CFUs of lactobacilli, respectively, *via* oral gavage; group 7 was injected with phosphate-buffered saline (PBS); group 8 remained untreated (UN). On days 14 and 21 post-hatch, birds were immunized intramuscularly with 100 μg KLH in 0.25 ml PBS. The untreated group (UN) was inoculated intramuscularly with 0.5 ml of PBS and served as the negative control. Statistical significance among treatment groups was calculated using SAS Proc GLM (General Linear Model) followed by Tukey’s comparison test. Error bars represent standard errors of the mean. Results were considered statistically significant if *P* < 0.05. Bars with asterisks represent a significant difference among treatments. The data are representative samples from 12 individual birds.

The results for anti-KLH IgM responses are presented in **Figure 8A**. On days 7, 14 and 21 post-primary immunization, higher anti-KLH IgM titers were observed in all immunized groups as compared to the negative control group (non-treated and non-immunized birds, P < 0.05). Birds that received 10^7^ CFU lactobacilli *via in ovo*, gavage, and both delivery routes (Groups 4) showed higher levels of anti-KLH IgM titers on day 7 post-primary immunization when compared to the positive control group (*in ovo* injected with PBS and immunized with KLH, P < 0.05). Higher anti-KLH IgM titers were observed in the group that received 10^7^ CFU lactobacilli *via* both delivery routes on day 14 days post-primary immunization (P < 0.05).

The results for anti-KLH IgY responses are presented in **Figure 8B**. On days 7, 14 and 21 post-primary immunization, higher anti-KLH IgY titers were observed in all immunized groups as compared to the negative control group (non-treated and non-immunized birds, P < 0.05). On day 21 post-primary immunization, the birds that received lactobacilli *via* both delivery routes (groups 3 and 4) showed higher anti-KLH IgY titers compared to positive control group (*in ovo* injected with PBS and immunized with KLH, P < 0.05); in addition, oral administration of 10^7^ CFU of lactobacilli increased anti-KLH IgY titers compared to the positive control group (P < 0.05).

## Discussion

Considering the developing state of the immune system in neonatal chickens, early establishment of gut microbiota with beneficial microbes may reduce the risk of post-hatch infection (22, 28). There is evidence that intestinal colonization with beneficial bacteria, including lactobacilli can modulate innate responses as well as cell- and antibody-mediated immune responses to subsequent antigen inoculation (21, 29, 30). In the present study, we evaluated the effect of early colonization of the chicken intestine with lactobacilli on the development of immune competence in newly hatched chicks.

Given the important role of cytokines in the immune system activation (31), the expression of cytokine genes in lymphoid organs of chickens was assessed following *in ovo* and oral administration of lactobacillus-based probiotic candidate. In general, combined administration of 10^7^ CFU of lactobacilli induced overall greater cytokine and chemokine expression in spleen and bursa of Fabricius than did either administration method alone. Noticeably, in birds that received 10^7^ CFU/egg of lactobacilli *via* both routes, significantly higher expression of IFN-α, IFN-β and IL-13 was observed in the spleen and significantly higher expression of IFN-γ, IL-2, IL-6, IL-8, IL-12, and IL-18 was observed in the bursa of Fabricius on day 10 post-hatch. These findings indicate that the number of lactobacilli as well as the route and frequency of administration are crucial to generate robust immune responses. In line with these observations, Brisbin and colleague (16) demonstrated that oral treatment of chickens with 10^7^ CFU of lactobacilli (*L. salivarius*, *L*. *acidophilus*, *L*. *reuteri*) increased expression of IFN-γ and IL-12 in splenic mononuclear cells (16). Wang and colleagues (32) also showed that *L. plantarum* P-8 enhanced the expression of T helper (Th)-type 1 and Th-type 2 cytokines in the small intestine of chickens (32). In another study, Taha-Abdelaziz and colleagues (33) investigated the effects of different *Lactobacillus* strains (*L. salivarius*, *L*. *johnsonii*, *L*. *reuteri*, *L. crispatus*, and *L*. *gasseri*) on cytokine gene expression using a chicken macrophage cell line, with results showing that all *Lactobacillus* isolates, either alone or in combination, increased the expression of IFN-γ, IL-1β, IL-12p40, and IL-10 (33).

In the present study, irrespective of the number of lactobacilli, *in ovo* administration of lactobacilli downregulated the expression of IFN-β, IFN-γ, TGF-β and IL-18 in the spleen but had no effect on cytokine and chemokine expression in the bursa of Fabricius. A previous study by our group demonstrated that *in ovo* lactobacilli supplementation did not alter the expression of cytokines in the bursa of Fabricius; additionally, it was shown that expression of pro-inflammatory cytokines was downregulated in the cecal tonsils of lactobacilli-treated birds (21). It should be noted that in the current study, cytokine and chemokine expression was measured only on days 5 and 10 post-hatch. Considering that cytokines and other immune system genes are inducible and transiently expressed, it is plausible that expression of some genes studied here (especially in in ovo groups) might have been altered at some point prior to the sampling time point. Thus, future studies are required to capture the expression of these genes at earlier time points.

Macrophages play a key role in host immune defense through phagocytosis of pathogens and activation of lymphocytes by processing and presenting antigens to T lymphocytes (34, 35). In the present study, *in ovo* administration of 10^7^ CFU of lactobacilli increased the number of KUL01^+^ cells in the spleen on day 5 post-hatch, while no significant difference was observed in chickens that received lactobacilli through both *in ovo* and oral routes. This likely indicates the importance for early inoculation of lactobacilli that can differentially affect macrophage numbers and function. As such, higher numbers of lactobacilli or more frequent administration of lactobacilli may not always lead to enhanced immune responses. In agreement with our finding, a previous study demonstrated that a *Lactobacillus*-based probiotic culture significantly increased the number of macrophages in the intestine of chickens (36). Therefore, promoting pre-hatch colonisation of lactobacilli can in fact affect mucosal responses to subsequent stimulation.

In the avian immune system, CD4^+^ T cells play a key role in adaptive immunity by activation of B cells in addition to their role in the induction and recruitment of macrophages to the site of infection (37). In the present study, the groups that received 10^7^ CFU of lactobacilli *in ovo*, and those that received combined administration (10^7^ CFU), had higher numbers of CD4^+^ cells compared to the control. The notable increase in CD4^+^ T cells population following *in ovo* and post-hatch lactobacilli administration indicates that these bacteria could promote the development of lymphoid organs, thus potentially improve resistance of young chickens to microbial pathogens. In agreement with these results, Asgari and colleagues (38) demonstrated that dietary supplementation with *L. acidophilus* (10^9^ CFU/kg) significantly increases the percentage of CD4^+^ T cells in chickens’ peripheral blood (38). Bai and colleagues (39) have also demonstrated that dietary supplementation of *L. fermentum* significantly increases proportions of CD3^+^ and CD4^+^ T lymphocytes in intestinal intraepithelial lymphocytes (IELs) of chickens (39). Another study demonstrated that oral treatment with *L. reuteri, L. salivarius* and *L. acidophilus* increased the number of CD4^+^ T lymphocytes in chicken intestine (40). Interestingly, despite their ability to increase the number of CD4^+^ T cells, the number of cytotoxic CD8^+^ T cells was not affected by any of the concentrations or delivery routes of lactobacilli used in this study. These results are inconsistent with previous studies that demonstrated that lactobacilli supplementation significantly increases CD8^+^ T lymphocytes in IELs and spleen (38–40). This could be explained by differences in strains and concentration of lactobacilli used in these studies.

Similar to the results obtained for CD4^+^ T cells, *in ovo* administration of 10^7^ CFU of lactobacilli, either as a single dose or combined with post-hatch oral treatment, increased the number of CD4^+^CD25^+^ T cells. In chickens, CD4^+^ CD25^+^ T cells have been shown to exhibit immunoregulatory properties by secreting immunosuppressive cytokines such as IL-10 and TGF-β that limit inflammatory response towards the end of the inflammatory processes (41). Therefore, the higher number of CD4^+^CD25^+^ T cells observed in lactobacilli-treated group supports the role of probiotic lactobacilli in maintaining the immune system homeostasis (42, 43). Results from some human studies suggest a role for lactobacilli in the modulation of monocyte-derived dendritic cells (DCs) and the development of Treg cells (44, 45). However, the exact mechanism by which these lactobacilli affect T cells is still unclear. It has been suggested that some lactobacilli (such as *L. reuteri* and *L. casei)* modulate monocyte-derived DCs by binding to the lectin DC-specific intercellular adhesion molecule 3-grabbing nonintegrin which triggers DCs to induce the development of IL-10-producing Treg cells.

While probiotic lactobacilli demonstrated the ability to increase the numbers of different T cell subsets, they do not seem to demonstrate such effects on Bu-1^+^ IgM^+^ B cell populations in the spleen. In agreement with our results, previous studies showed that various *Lactobacillus* isolates had no effects on Bu-1 mRNA expression levels (39, 46). It was suggested that lactobacilli modulate the immune system mainly through interaction with T lymphocytes rather than B cells, implicating a T-dependent B cell activation mechanism.

In addition to their role in eliciting innate and cell-mediated immune responses, probiotic lactobacilli have shown to enhance antibody-mediated responses to various antigens (47–49). KLH and SRBC are often used as gold standards to assess B cell functional responsiveness due to their non-toxic nature as a xenogeneic antigen. Both SRBC and KLH are considered thymus-dependent antigens, which require the cooperation of T helper cell for B cell activation and proliferation (50). The results of the current study demonstrated that *in ovo* administration of 10^7^ CFU of lactobacilli, either as a single dose or combined with post-hatch oral treatment, significantly enhanced antibody responses to SRBC, suggesting the importance of early inoculation of lactobacilli in adaptive immune response activation. With respect to anti-KLH antibody titers, the group that received the combination of *in ovo* and weekly oral administration of lactobacilli (10^7^ CFU) consistently increased IgM titers on days 7 and 14- and IgY titer on day 21 post-primary immunization. These results are consistent with our earlier findings that oral and *in ovo* administration of lactobacilli enhanced IgY and IgM antibody responses against KLH (16, 21). Collectively, the notable increase in SRBC and KLH antibody responses following treatment with lactobacilli is indicative of adjuvant properties of probiotic lactobacilli. The exact mechanisms underlying this effect remains to be elucidated. It could be attributed to their interactions with pattern recognition receptors (expressed by cells of innate immune system) and subsequent production of cytokines (IL-4, IL-10, and IL-13) involved in B cell development and antibody production (51).

In conclusion, the results of the present study demonstrate that inoculation with a mixture of lactobacilli *via* the *in ovo* route or early post-hatch administration *via* oral gavage could trigger innate and adaptive immune response. Administration of lactobacilli could also enhance the development of lymphoid organs, providing early protection to hatchlings. More importantly, overall improved immune responses were attained when these lactobacilli were administered in *ovo* followed by weekly oral administration to hatched chicks. Further studies are required to evaluate the effects of these lactobacilli on the development of gut-associated lymphoid tissues and other lymphoid organs and assess their protective efficacy against infectious agents.

## Data Availability Statement

The raw data supporting the conclusions of this article will be made available by the authors, without undue reservation.

## Ethics Statement

The animal study was reviewed and approved by Animal Care Committee, University of Guelph.

## Author Contributions

MA and SS designed the experiment. MA performed the experiment, collected and analyzed the data, and wrote the first draft of the manuscript. JB, BS, JA, KT-A, NA, NB, and JS helped for samples collections, processing, and analysis, reviewed the manuscripts, and provided suggestion and comments. SS provided intellectual input, approved the protocol, critically reviewed the manuscript, and provided suggestion, and comments. All authors contributed to the article and approved the submitted version.

## Funding

Funding was partly provided by the Ontario Ministry of Agriculture Food and Rural Affairs, NSERC, and the Canadian Poultry Research Council. This research is supported in part by the University of Guelph’s Food from Thought initiative, thanks to funding from the Canada First Research Excellence Fund.

## Conflict of Interest

JA was employed by the company Artemis Technologies Inc.

The remaining authors declare that the research was conducted in the absence of any commercial of financial relationship that could be construed as potential conflict of interest.
