# Corrigendum: *In ovo* and oral administration of probiotic lactobacilli modulate cell- and antibody-mediated immune responses in newly hatched chicks

**DOI:** 10.3389/fimmu.2023.1252523

**Published:** 2023-08-08

**Authors:** Mohammadali Alizadeh, Jegarubee Bavananthasivam, Bahram Shojadoost, Jake Astill, Khaled Taha-Abdelaziz, Nadiyah Alqazlan, Nitish Boodhoo, Janan Shoja Doost, Shayan Sharif

**Affiliations:** ^1^ Department of Pathobiology, Ontario Veterinary College, University of Guelph, Guelph, ON, Canada; ^2^ Department of Pathology and Molecular Medicine & McMaster Immunology Research Centre, M. G. DeGroote School of Medicine, McMaster University, Hamilton, ON, Canada; ^3^ Quality Control Department, Artemis Technologies Inc., Guelph, ON, Canada; ^4^ Department of Pathology, Faculty of Veterinary Medicine, Beni-Suef University, Beni-Suef, Egypt

**Keywords:** lactobacilli, chicken, cytokine, lymphocyte, antibody, immune response

**Error in Figure/Table**

In the published article, there was an error in **Table 2** as published. The mistake occurred during copying and pasting the sequences of primers, and so the wrong primer sequences were inserted. Corrected **Table 2** and its caption appear below.

**Table 2:** Primer sequences used for real-time quantitative PCR^1^.

Gene^2^	Primer sequence^3^ (5’-3’)	Annealing temperature	GeneBank accession number
*IFN-*α	F: ATCCTGCTGCTCACGCTCCTTCT R: GGTGTTGCTGGTGTCCAGGATG	64	AB021154
*IFN*-β	F: GCCTCCAGCTCCTTCAGAATACG R: CTGGATCTGGTTGAGGAGGCTGT	64	AY974089
*IFN-*γ	F: ACACTGACAAGTCAAAGCCGCACA R: AGTCGTTCATCGGGAGCTTGGC	60	X99774
*IL-1*β	F: GTGAGGCTCAACATTGCGCTGTA R: TGTCCAGGCGGTAGAAGATGAAG	64	Y15006
*IL-2*	F: TGCAGTGTTACCTGGGAGAAGTGGT R: ACTTCCGGTGTGATTTAGACCCGT	60	NM_204153.2
*IL-6*	F: CGTGTGCGAGAACAGCATGGAGA R: TCAGGCATTTCTCCTCGTCGAAGC	60	NM_204628.1
*IL-8*	F: CCAAGCACACCTCTCTTCCA R: GCAAGGTAGGACGCTGGTAA	64	AJ009800
*IL-12p40*	F: CCAAGACCTGGAGCACACCGAAG R: CGATCCCTGGCCTGCACAGAGA	60	AY262752.1
*IL-13*	F: ACTTGTCCAAGCTGAAGCTGTC R: TCTTGCAGTCGGTCATGTTGTC	60	AJ621250.1
*IL-18*	F: GAAACGTCAATAGCCAGTTGC R: TCCCATGCTCTTTCTCACAACA	64	AY628648.2
*TGF-*β*^4^*	F: CGGCCGACGATGAGTGGCTC R: CGGGGCCCATCTCACAGGGA	60	M31160.1
β*-Actin*	F: CAACACAGTGCTGTCTGGTGGTA R: ATCGTACTCCTGCTTGCTGATCC	58	X00182

^1^The listed oligonucleotides were used to analyze gene expression via real-time quantitative PCR.

^2^IFN, Interferon; IL, Interleukin.

^3^F, forward; R, reverse.

^4^TGF-b, transforming growth factor beta.

The authors apologize for this error and state that this does not change the scientific conclusions of the article in any way. The original article has been updated.

